# Organizational Aspects of the Implementation and Use of Whole Genome Sequencing and Whole Exome Sequencing in the Pediatric Population in Italy: Results of a Survey

**DOI:** 10.3390/jpm13060899

**Published:** 2023-05-26

**Authors:** Mario Cesare Nurchis, Gian Marco Raspolini, Aurora Heidar Alizadeh, Gerardo Altamura, Francesca Clementina Radio, Marco Tartaglia, Bruno Dallapiccola, Gianfranco Damiani

**Affiliations:** 1 Department of Woman and Child Health and Public Health, Fondazione Policlinico Universitario A. Gemelli IRCCS, 00168 Rome, Italy; nurchismario@gmail.com (M.C.N.); gianfranco.damiani@unicatt.it (G.D.); 2 School of Economics, Università Cattolica del Sacro Cuore, 00168 Rome, Italy; 3 Department of Health Sciences and Public Health, Section of Hygiene, Università Cattolica del Sacro Cuore, 00168 Rome, Italy; aurora.heidar@gmail.com (A.H.A.); gerardoaltamura@outlook.it (G.A.); 4 Molecular Genetics and Functional Genomics, Ospedale Pediatrico Bambino Gesù IRCCS, 00146 Rome, Italy; fclementina.radio@opbg.net (F.C.R.); marco.tartaglia@opbg.net (M.T.); bruno.dallapiccola@opbg.net (B.D.)

**Keywords:** pediatric population, whole genome sequencing, whole exome sequencing, organizational issues, survey

## Abstract

This study explores the organizational aspects of whole genome sequencing (WGS) implementation for pediatric patients with suspected genetic disorders in Italy, comparing it with whole exome sequencing (WES). Health professionals’ opinions were collected through an internet-based survey and analyzed using a qualitative summative content analysis methodology. Among the 16 respondents, most were clinical geneticists performing only WES, while 5 also used WGS. The key differences identified include higher needs for analyzing genome rearrangements following WES, greater data storage and security requirements for WGS, and WGS only being performed in specific research studies. No difference was detected in centralization and decentralization issues. The main cost factors included genetic consultations, library preparation and sequencing, bioinformatic analysis, interpretation and confirmation, data storage, and complementary diagnostic investigations. Both WES and WGS decreased the need for additional diagnostic analyses when not used as last-resort tests. Organizational aspects were similar for WGS and WES, but economic evidence gaps may exist for WGS in clinical settings. As sequencing costs decline, WGS will likely replace WES and traditional genetic testing. Tailored genomic policies and cost-effectiveness analyses are needed for WGS implementation in health systems. WGS shows promise for enhancing genetics knowledge and expediting diagnoses for pediatric patients with genetic disorders.

## 1. Introduction

Rare diseases (RDs) are defined on the basis of their low prevalence in the general population. There are more than 10,000 known RDs presently, affecting about 300 million individuals worldwide [1]. It is estimated that 50–70% of RDs present a childhood onset, and among all children affected, 25% will die before the age of five. The symptomatology of RDs can be particularly complex and nuanced. Most RDs are still not curable, yet a timely and accurate diagnosis followed by appropriate medical care can prolong the life expectancy and improve the quality of life of affected individuals and their families [2]. Next-generation sequencing (NGS) techniques, such as whole-genome and whole-exome sequencing (WGS and WES), allow to identify pathogenic variants, examining, respectively, the entire genome and targeted fragments of interest (i.e., the coding exons of approximately 20,000 genes). These genetic tests find significant applications in diagnosing RDs of intensely ill children and newborns, particularly by aiding the implementation of a targeted care pathway and improving the efficacy of their clinical management [3]. It has been shown that WGS gives a better diagnostic yield compared to WES; notwithstanding, the use of WGS is still limited to research purposes in Italian facilities [4]. Moreover, there is still little evidence supporting the implementation of WGS in clinical contexts, as outlined by Health Technology Assessment (HTA) reports, which are dedicated tools for the multidisciplinary evaluation of a medical technology [5]. Among the elements to be considered in non-clinical domains of HTA reports, organizational aspects are crucial and should be explored thoroughly. In fact, they represent the domain in which information needs to be both efficiently synthesized and appropriately described, to guide stakeholders in the decision-making process regarding the assessed technology. Previous studies explored other elements of WGS in children, such as perceived knowledge and attitudes of healthcare professionals towards its inclusion in clinical practice [6], insights for developing adequate interventions leading to its implementation in the pediatric/neonatal critical care [7], concerns and opinions regarding its risks/benefits, the barriers of its clinical integration [8], and the interest of parents in WGS [9].

The aim of this study was to investigate the organizational aspects of WGS implementation, compared to WES, relative to its inclusion in the Italian clinical context and the diagnostic workflow for pediatric patients with suspected genetic disorders.

## 2. Materials and Methods

### 2.1. Study Design

An internet-based survey was performed to assess the organizational aspects of WGS, with respect to WES, for the pediatric population in the Italian context.

### 2.2. Recruitment

Health professionals involved in the diagnostic workup of children with suspected genetic disorders were contacted through Italian professional associations and requested to fill out the survey with the aim of guiding stakeholders in decision making about WGS and/or WES. The target population consisted of experts working in tertiary care facilities across Italy for a total of 20 centers, including clinical geneticists, biologists, bioinformaticians, and other professionals. The population heterogeneity was intended to reach comprehensiveness in the assessment of the organizational aspects of these technologies. The sample size was estimated by data saturation. Additional details are provided in the Data Analysis section.

### 2.3. Questionnaire Development

The structured questionnaire was elaborated according to previous evidence [10,11] exploring the organizational aspects of NGS technologies in an HTA perspective, and in line with EUnetHTAs indications [12]. The questionnaire was based on a facilitated framework proposed by Cacciatore et al. [13] exploring how organizational assessment is carried out in HTA reports. Out of the fifteen issues listed in the Core Model’s “ORG” domain [12], four linked by Cacciatore et al. to diagnostic technologies such as WGS and WES (i.e., G0004, G0005, G0006, and D0023) were investigated. The structured questionnaire included five sections addressing the respondents’ views and perspectives on the following aspects: (1) characteristics of participants and their clinical centers; (2) mobilization of co-operation and communication activities; (3) influence of centralization or de-centralization on the implementation of WGS and WES; (4) costs of acquisition and setting up the technology; and (5) the need for other technologies and resources while implementing WGS and WES.

In the second section (i.e., “0004: Which type of co-operation and communication activities need to be mobilized?”), respondents were asked to describe the steps characterizing the WGS workflow and the actors involved in each step. In the third section (i.e., “G0005: How do decentralization or centralization requirements influence the implementation of the technology?”), respondents were asked to report whether the implementation of WGS and WES is influenced by organizational requirements related to the decentralization or centralization of specific activities. In the fourth section (“G0006: Which are the costs of processes related to acquisition and setting of the new technology?”), participants were asked to detail the main cost items associated with the WGS and WES. In the fifth section (“D0023: How does the technology modify the need of other technologies and use of resources?”), participants were requested to specify if the implementation of WGS and WES requires other technologies or resources. 

The questionnaire was drafted and made available to the participants in Italian. It was not password protected and was developed and disseminated using Google Forms online survey software.

### 2.4. Data Collection

The first invitation was sent in December 2022, and the questionnaire was available until the end of February 2023. A reminder was sent four weeks after the opening invitation of the survey. Both these emails requested the participants to share the link to the questionnaire with other health professionals in their institutions involved in the workflows of WGS and/or WES. Therefore, due to snowball recruitment procedures and dissemination by invited health professionals, the authors were unaware of the exact total number of participants.

Institutional Research Ethics Board approval was sought for this survey but ruled not to be required under the Italian legislation. Informed consent was obtained from participants before they started the questionnaire. Participants were informed about the purpose of the study. Participation in the survey was voluntary with the possibility to opt-out at any moment. No incentives were offered for completion of the questionnaire. Data collection was anonymous from the researchers’ standpoint.

### 2.5. Data Analysis

Data saturation is one of the most adopted approaches for computing sample sizes in qualitative research [14,15,16]. Saturation refers to “the point at which gathering more data about a theoretical construct reveals no new properties, nor yields any further theoretical insights about the emerging grounded theory” [17]. Following the method suggested by Guest et al. [14], saturation was estimated using a base size of four, a run length of two, and a new information threshold of ≤5% to highlight adequate saturation.

Researchers first assessed the survey completeness for each participant, and questionnaires with missing information were excluded from analyses.

The responses from all participants were entered into an electronic spreadsheet manually, whose content was verified for correctness by three investigators. 

A descriptive analysis of the respondent’s demographics and center characteristics was performed, adopting frequencies and standard deviations. Furthermore, a qualitative analysis was conducted examining free-text responses to the open-ended questions using the summative thematic analysis [18], thus identifying keywords and exemplar quotes for themes related to the organizational aspects dealing with the implementation in the clinical practice of WGS in respect to WES.

## 3. Results

### 3.1. Participants’ and Institutions’ Characteristics

Saturation of data was reached at the 13th survey. The demographics of the 16 respondents are reported in Table 1. A total of 75% of respondents were physicians, 83% of which were geneticists and the remaining 17% were pediatricians and resident medical geneticists. Most of the latter were clinical geneticists (*n* = 11), the remaining being laboratory geneticists (*n* = 5). We asked the participants which NGS technique was performed in their centers. All reported the use of WES, and only five reported the use of WGS as well. Almost all respondents worked in publicly owned centers (*n* = 12). One third of the participants worked in institutions located in the Lazio region, followed by those in Lombardy (19%), Emilia-Romagna (13%), Piedmont (6%), Friuli-Venezia Giulia (6%), Liguria (6%), Campania (6%), Calabria (6%), and Apulia (6%).

### 3.2. Health Delivery Process

Health professionals were asked to describe the steps needed to perform the diagnostic procedure through WGS. The steps were identified and reported as follows.

A medical specialist (e.g., pediatrician, neurologist, pediatric surgeon, or cardiologist) asks for a genetic consultation to obtain a diagnosis for a patient affected by a likely genetic disease. The request may be complemented by a document reporting a description of said clinical condition, possibly filled out according to the internationally recognized disease ontologies. Afterwards, a medical geneticist performs the genetic consultation and, if specific sets of criteria are fulfilled (e.g., clinical and demographic criteria), informed consent to perform WGS for defined research purposes is sought and obtained, which enables the enrollment of the patient, and possibly their parents, in a study. Then, blood or other tissue samples from the enrolled individuals are collected and sent to the laboratory. In some cases, the laboratory is located in a different facility. The first steps in the laboratory include DNA extraction, purification, and quality assessment. Nucleic acid is quantified and eventually diluted. At this stage, further purification of the material is carried out if necessary. Library preparation and sequencing are then performed. Sequence data are generated and stored in given file formats (e.g., FASTQ, FASTA, SAM, BAM, and VCF). When a different facility is responsible for the variant calling process, the files are sent to it. The processes of variant calling, annotation, and prioritization are performed according to locally implemented pipelines to the patient’s phenotype and other data (e.g., minor allele frequency and in silico tools predicting functional relevance and impact). Identified variants are reported to the clinical geneticist/pediatrician, who reassesses the patient based on the WGS results. The variants suspected to be responsible for the patient’s clinical manifestations are validated using a segregation analysis and Sanger sequencing. Diagnostic hypotheses might be further discussed among clinicians. A clinical report is produced and the results are explained to the patient and/or their parents or caregivers. Stored genomic data can be reanalyzed.

The main actors involved in the above process can be grouped based on the following activities: sample collection, laboratory activities, clinical case discussion, and communications with families and/or caregivers. Samples are collected by nurses.

Laboratory activities are generally managed by geneticists in charge of the clinical case and interpretation of sequencing data and other professionals, such as bioinformaticians for analyses, laboratory technicians for management of practical tasks (i.e., DNA extraction and data validation), biologists for the use of bioinformatic pipelines, and administrative personnel. The laboratory geneticists also interface with other medical specialists involved in the management of the specific clinical case.

Medical specialists and biologists are involved in the clinical case discussion, which comprises the choice of bioinformatic pipelines, interpretation of variants, assessment of results, clinical implications, and addressing the potential need for further investigations. Communications with patients, parents, and caregivers are carried out by physicians and jointly with psychologists if necessary. 

**Figure 1:**
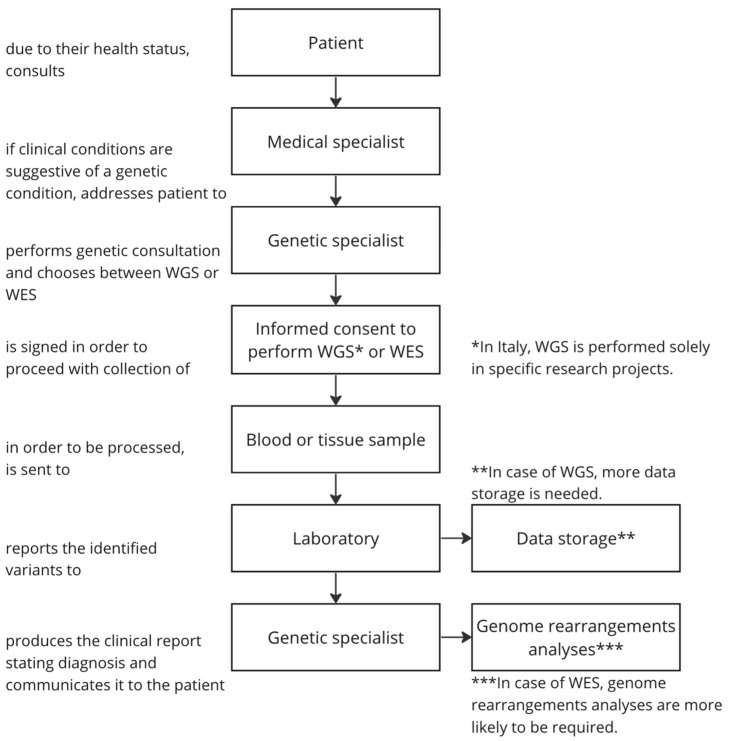
A flow diagram representing the steps of the diagnostic workflow involving WGS or WES use in Italy. The asterisks identify the phases which were significantly different between WGS and WES according to the responders’ answers.

By comparing WGS and WES in terms of required steps, involved actors, and related activities, the differences emerging from the responders included (1) a deeper analysis of genome rearrangements after WES, (2) major requirements for storing and securing data for WGS, and (3) possibility of performing WGS only in selected individuals enrolled in research studies. Figure 1 depicts the WGS and WES workflow based on the analysis of the responders’ answers.

Responders were asked to outline any collaboration and/or interaction at national and/or international levels with centers using WES and/or WGS. Participants from the Lazio region reported that collaboration was mainly based on specific research activities, in-depth medical investigations, and know-how exchange. Collaborative activities of respondents working in Lombard centers entailed the discussion of complex cases, as well as research activities on gene discovery and interpretation of VUS, including the collaboration within the European Reference Networks. Responders from the Emilia-Romagna region reported collaborations with the Gaslini Institute and the Center for Human Technologies in Genoa, with Fondazione IRCCS Policlinico San Matteo in Pavia in the context of research projects, as well as in the Epi25 international network on epilepsy. Participants from the Friuli-Venezia Giulia region reported active involvement in a study dealing with the identification of genetic risk factors for cardiovascular diseases and the molecular background of neurodevelopmental disorders, and several international collaborations (e.g., ReproGen consortium). Respondents from the Calabria region reported close collaboration with the Ospedale Pediatrico Bambino Gesù in Rome, including discussions of complex patients and reanalysis of undiagnosed patients’ genomic data using shared pipelines. Centers located in the Liguria region reported collaboration with the Italian Institute of Technology to which library preparation, sequencing, and some bioinformatic tasks are outsourced. Responders from the Piedmont region highlighted a public–private cooperation with 3billion, Inc. for scientific research activities. The Campania region has at least one publicly owned center using only WES, which works jointly with Ospedale Pediatrico Bambino Gesù. The Apulia region responders reported that their lab has not established yet any collaboration with other centers.

The main points emerging from this section are summarized in Table 2.

### 3.3. Structure of the Health Care System

In this section, respondents were asked to report the steps of the WGS/WES diagnostic workflow they deemed appropriate to be feasible in secondary or primary care facilities in order to achieve the greatest patient accessibility to the technology and the best quality of care. Out of sixteen participants, only two respondents considered the decentralization of some steps of the diagnostic workflow convenient, while this percentage increased to 40% when only the use of WGS was considered. One argued for the usefulness of decentralizing the identification of patients eligible for WGS and WES and the sharing of clinical management to ease continuity of care and improve literacy and awareness on rare diseases. The other respondent claimed that, both for WGS and WES, genetic counseling should be performed by dedicated specialized personnel, which should operate also in secondary care centers. The respondent suggested also that the laboratory and bioinformatic processes should be centralized and carried out exclusively in tertiary care facilities, given the economic and organizational issues. Lastly, another highlighted organizational issue was related to the storage and securing of genomic data, as they may be useful to be analyzed repeatedly for clinical and research purposes.

The main themes and subthemes of this section are summarized in Table 3.

### 3.4. Process-Related Costs

Responders were requested to list and share information about the costs of WGS and/or WES at different stages of the implementation process. Six participants (38%) provided evidence from the scientific literature about health economic evaluations of WGS and WES implementation in the diagnostic workflow for pediatric children with suspected genetic disorders. These studies identified genetic consultations, library preparation and sequencing, bioinformatic analysis, interpretation and confirmation, data storage and securing, and other complementary diagnostic tests as the main costs of short-read NGS technique implementation. The detailed characteristics of the scientific articles suggested by the responders are summarized in Table S1 in the Supplementary Material. One participant (6%) reported that the estimation of WES/WGS costs is performed by their hospital management control but did not provide any detailed information. Half of responders (56%) did not fill this part of the questionnaire. 

### 3.5. Resource Utilization

Participants were asked to what extent availability of WGS/WES in their centers influenced the use of other genetic tests. The participant working in Friuli-Venezia Giulia wrote that these possibilities drastically decreased the use of other genetic tests. Similarly, in a publicly owned center in Lombardy, a low a priori probability of obtaining a diagnosis with a single targeted test and the urgency of solving the diagnosis according to the children’ clinical conditions are considered the key aspects favoring the use of WGS as first-tier test, thus avoiding the exploitation of other genetic technologies and resources.

A respondent from a private institution in Lazio stated that, in specific research projects, WGS is either executed on undiagnosed patients after negative targeted genetic tests, clinical and/or research WES, or used as a first-tier assay in patients with complex phenotypes of likely genetic origin. In the same center, access to WES reduced the need for single-gene and panel tests, thus improving the diagnostic yield. A clinical geneticist and a biologist, working in public institutions in Lazio and Emilia Romagna, respectively, claimed that patients with complex disorders underwent WGS after previous inconclusive genetic tests. This implies that, in these two centers, the possibility of using WGS, adopted as a last choice, did not influence the use of other genetic tests.

Respondents from the centers in Piedmont, Campania, Lombardy, and Apulia, in which only WES was available, agreed that the use of this technique drastically reduced the number of other genetic tests, while responders from Lazio and Liguria stated that WES is performed as the last option.

Participants were also asked if a widespread use of WGS could simplify the diagnostic pathway in patients with suspected genetic diseases, and, if so, to explain the rationale for this simplification. Most respondents (75%) answered affirmatively and reported that WGS would cover most of the genetic abnormalities and increase the probability of obtaining a diagnosis with a single genetic test under tight time constraints, despite current WGS interpretative hurdles. An additional reason for preferring WGS was the possibility to reanalyze the patients’ genome based on new clinical findings and genetic advances. 

The respondents working in centers where only WES was available suggested that the implementation of WGS increased the efficacy of reporting, allowed the adoption of a one-shot analysis approach, and progressively abandoned sequencing confirmation techniques, consequently increasing automation. Referring to WES, all responders suggested that a more extensive use of this technique would allow to diagnose a large population of undiagnosed patients, shorten the diagnostic time, and reduce the use of other tests. A responder from Calabria also suggested, as additional benefits, decreased public expenditure for additional tests, a reduction in medical tourism and its related costs, the sharing of patient data that results in gains in therapeutic compliance, building public trust and satisfaction, and improved quality of care.

Finally, responders were asked to point out which patients would benefit the most from WGS as a first-tier genetic test. More than half of the respondents (56%) suggested undiagnosed pediatric patients with complex phenotypes, such as those admitted to NICU/PICU. Twenty-six percent of the responders stated that WGS would be the first choice in prenatal genetic diagnostics. A minority of participants (18%) did not consider WGS a workable first-line genetic test. The same targeted population was also considered suitable for WES.

The main points identified in this section are summarized in Table 4. 

## 4. Discussion

This survey refers to Italian health professionals’ views of the organizational aspects regarding the implementation and use of WGS and WES for pediatric patients. The survey included a broad range of professionals involved in the workflows of either one of the two tests or both. Overall, no differences between WGS and WES in terms of the actors involved in communication and cooperation activities emerged from the survey. The significant differences pertain to the steps required for carrying out the two protocols. In particular, WES steps following sequencing more often require dedicated analyses directed to investigate structural rearrangements, which is rarely necessary in the case of WGS [19]. The storing and security issues are different for WGS compared to WES, given the much larger size of data files, ranging from hundreds of gigabytes to terabytes, required for examining entire genome sequenced alignment data [20]. WGS is performed exclusively in individuals enrolled in selected research studies, which may be explained by the fact that, as of today, the technique is not listed among the Essential Levels of Care (Livelli Essenziali di Assistenza, LEA) [21], rendering it not reimbursable by the Italian National Health Service (NHS).

WGS detects a higher number of structural variants compared to WES, including large and rare variants missed by exome sequencing. Plesser et al. underlined that when the disruption responsible for a given disease affects an intronic region, WES does not detect the genetic defect, unlike WGS [22]. Schuy et al. reported that WGS, by producing longer genome reads, allows the comprehension of particularly complex genomic rearrangements, bringing to light a higher prevalence of this type of variants than hitherto believed [19].

Concerning WGS storage and security issues, Tanjo et al. suggested that the terabytes of data produced [20] will become easily and securely stored as new cloud systems technology is implemented [23].

The lack of reimbursement for WGS tests, which explains its exclusive use in the research context, could constitute a significant deprivation for clinicians.

The survey has stressed that strong collaboration does exist between centers using NGS techniques, and it mainly focuses on research activities, in-depth medical investigations, and know-how exchange. The scientific literature also confirms that collaboration is of paramount importance for WGS in genetic diseases, allowing the researchers to share resources and expertise, data and samples, and to work conjointly in order to identify the defects underlying genetic diseases more quickly and accurately [24,25].

Most of the responders emphasized the problem of decentralizing some steps of the diagnostic workflow, an inconvenience that, conversely, did not occur in centers using both WGS and WES and was instead regarded as useful. In line with this finding, Meneghini et al. demonstrated the feasibility of outsourcing some services in genetics (e.g., genetic consultations) [26]. Notably, the decision to decentralize WGS services for suspected genetic disorders may depend on several factors, including costs, quality, accessibility, and single center expertise.

Moreover, the results of our study indicate that the costs associated with implementing WGS and WES technologies are highly dependent on the specific steps involved in the diagnostic workflow. A possible explanation for this may be the larger amount of data produced by WGS [20] and its processing. As a result, data storage and analysis demand significantly more resources, with a consequent cost increase [27]. This is in line with studies supporting the cost-effectiveness of WES in comparison to the conventional genetic path [27,28,29,30]. In contrast to WGS, this technique usually needs to be completed by complementary tests, and thus with additional costs. Recent evidence supports the cost-effectiveness of first-line WGS compared to WES and standard diagnostic pathways for diagnosing infants and children with suspected genetic conditions [31,32].

The availability of WGS or WES can drastically reduce the use of other genetic tests in clinical settings, especially in cases where patients have complex phenotypes and no definitive diagnosis. Respondents agreed that the systematic use of WGS or WES as a first-tier test can improve the diagnostic yield, decrease the reporting time, and reduce public expenditure for additional tests. Furthermore, most of them believed that a wider adoption of WGS or WES could simplify the diagnostic pathway for suspected genetic diseases, covering most genetic abnormalities with a single genetic test and increasing the probability of reaching a diagnosis. In accordance with the present result, the clinical guidelines of the American College of Medical Genetics and Genomics (ACMG), and evidence from published papers, the adoption of WGS and WES as a first-tier test for broad pediatric populations is strongly recommended [33,34].

The joint reading of these findings allows for conceptual study implications. 

Communication and cooperation between centers performing WES and/or WGS are critical for advancing genomic medicine and improving patient care. Therefore, the scientific community may benefit from strengthening local, national, and international networks to identify, assess, support, and connect centers of expertise through a multi-disciplinary patient-centered approach. This can be particularly important for smaller centers with limited resources or expertise in genomic analysis, enabling them to benefit from the knowledge and resources of larger centers. In addition, the development of networks between centers and professionals could ease the de-centralization of some services and tasks by integrating skills, know-hows, and peculiarities of the main actors involved in the system.

Comprehensive cost-effectiveness analyses should be performed prior to introducing WGS or WES in a clinical setting. Such analyses should take into account the costs associated with both the acquisition and running of the diagnostic technology, as well as any required changes in hospital premises or infrastructures. The decision to introduce WGS or WES should be based on a thorough understanding of the costs and benefits, as well as the potential impact on patients’ outcomes. By considering these factors, healthcare organizations could make informed decisions about whether to adopt WGS or WES and how to optimize the implementation process to minimize costs and maximize patient outcomes. 

This study also raises the possibility of steering the decision-making process both at the macro-level, contributing to elaborate sound genomic policies and health programs focused on WGS, and at the micro-level, informing the development of guidelines for the appropriate use of WGS in clinical settings.

The present analysis must be considered in light of its main limitations and strengths. Firstly, the sample size is small (i.e., 16) and not every expert contacted replied to the questionnaire, leading to a potential selection bias. Notwithstanding, this qualitative study reached saturation at an even smaller sample size (i.e., 13), as also confirmed by evidence in the scientific literature [35,36]. Moreover, the inclusion and thus the invitation to complete the questionnaire was restricted to highly specialized experts working in tertiary care facilities in Italy, which are low in number across the country, using either one of the two tests in study or both. Furthermore, the low number of respondents might be ascribable to infrastructural barriers, cultural backgrounds, personal beliefs, and also the submission of considerably time-demanding questions. However, the professionals who actually responded to our survey are interested in the middleic and wanted their opinions to be heard, so their responses are reflected in the results. Besides, the choice of the proposed questions was grounded on a rigorous and internationally validated methodology, derived from the scientific literature. Another caveat is the limited heterogeneity in the responders’ occupation, i.e., only physicians and biologists. The view of other professionals, such as bioinformaticians and laboratory technicians, would have enriched the study findings. Nonetheless, biologists and especially physicians are the actors involved in most of the steps of the diagnostic workflow.

Similar research should be conducted investigating the policy and legal and social aspects of implementing NGS techniques for the pediatric population with suspected genetic conditions. Further studies are required to understand the points of view of professionals working with WGS in countries where this technology is already well established to obtain a comprehensive overview of the organizational aspects at the international level. Besides, additional research is needed to fully prove the feasibility and sustainability, in an HTA perspective, of implementing WGS in clinical practice.

## 5. Conclusions

According to the perception of the respondents, organizational aspects are largely comparable between WGS and WES. There could be gaps in sustainability and funding information in support of WGS in clinical settings. Nonetheless, considering the steady decline in sequencing costs, WGS might take the place of WES and traditional genetic testing [37].

Our results highlight the potential need for establishing sound genomic policies for strengthening collaborative working networks and performing rigorous cost-effectiveness analyses to prove the sustainability of WGS within health systems and to define a tailored reimbursement rate. The challenges of using WGS in clinical practice are substantial, but its wide application is promising, both for improving the understanding of human genetics and shortening the diagnostic journey of patients with suspected genetic disorders and their families.

## Figures and Tables

**Table 1:** Sample characteristics.

Variable	Estimate	SD
***Respondent gender***		
Female	9	0.51
Male	7	0.49
***Respondent age***		
25–34 years	6	0.50
35–44 years	1	0.25
45–54 years	2	0.34
55–64 years	7	0.51
***Respondent occupation***		
Physician	12	0.56
Biologist	4	0.41
***Respondent experience in the field of genetics and genomics***		
1–9 years	6	0.51
10–19 years	2	0.45
≥20 years	8	0.40

**Table 2:** Themes and illustrative quotes about the second section of the questionnaire.

Themes/Subthemes	Quotes
***Health delivery process***	
Steps in the diagnostic process	“The enrolled patients receive genetic consultation from a medical geneticist, who also seeks and obtains the informed consent to conduct WGS for research purposes.” (Physician)
Actors involved and actions required	“The laboratory geneticists communicate with other doctors who are handling the same clinical case, such as clinical geneticists, pediatricians, cardiologists, and neurologists.” (Physician)
Cooperation and communication activities	“Collaborative activities that involve other centers include discussion of complex clinical cases, research work on gene discovery, and interpretation of variants of uncertain significance.” (Physician)

**Table 3:** Themes and illustrative quotes about the third section of the questionnaire.

Themes/Subthemes	Quotes
***Structure of health care system***	
Role of decentralization	“Both for WGS and WES, genetic counseling should be performed by dedicated specialized personnel also in secondary care centers, more widely spread throughout the country.” (Biologist)
	“The economic and organizational aspects of these tasks require that laboratory and bioinformatics processes be centralized and performed only in tertiary care facilities.” (Physician)

**Table 4:** Themes and illustrative quotes about the fifth section of the questionnaire.

Themes/Subthemes	Quotes
***Resource utilization***	
Need for other diagnostics	“WGS and WES drastically decreased the use of other genetic tests in my center.” (Biologist)
Simplified diagnostic pathway	“[…] Despite current interpretation hurdles, whole-genome sequencing covers most genetic abnormalities such as SV and STR, increasing the likelihood of obtaining a diagnosis with a single genetic test under tight time constraints.” (Physician)
Eligible population	“Children with monogenic disorders characterized by locus heterogeneity in which a clinical exome analysis would likely result in negative or inconclusive findings.” (Physician)

## Data Availability

The data that support the findings of this study are available on request from the corresponding author, G.M.R.

## Supplementary Materials

The following supporting information can be downloaded at: https://www.mdpi.com/article/10.3390/jpm13060899/s1, Table S1: Characteristics of eligible scientific articles received regarding costs of WGS and WES.
