# Supplementary material for: Organizational Aspects of the Implementation and Use of Whole Genome Sequencing and Whole Exome Sequencing in the Pediatric Population in Italy: Results of a Survey
Source: J Pers Med. 2023 May 26;13(6):899. doi: 10.3390/jpm13060899 (PMC10304348; doi:10.3390/jpm13060899)
Supplement: Supplementary file 1 [file jpm-13-00899-s001.zip › jpm-2396036-supplementary.pdf]

Organizational aspects of the implementation and use of whole genome sequencing and whole exome sequencing in the pediatric population in Italy: results from a survey

Supplementary Material

Table S1. Characteristics of eligible scientific articles received regarding costs of WGS and WES.

**Author, year, country**	**Source**	**Study Type**	**Disease**	**Interventions and Comparator**	**Perspective**	**Economic Model**	**Costs**	**ICER (cost per additional diagnosis)**	**Results**
Tan TY, 2017, Australia	Jama Pediatrics	Cost-effectiveness analysis	Suspected Mendelian disorders	WES<br>SoC	Hospital	NA	WES: A$5,186.5<br>SoC: A$12,912.3	A$9020	Whole exome sequencing was most cost-effective when applied at initial presentation to tertiary care compared with first clinical genetics assessment and the standard diagnostic pathway.
Aaltio J, 2022, Finland	European Journal of Paediatric Neurology	Cost-effectiveness analysis	Neuorological diseases	WES<br>SoC	Hospital	NA	WES: €9,537<br>SoC: €9,910	Dominant	WES is an efficient and cost-effective diagnostic tool that should be prioritized in early diagnostic path of children

									with progressive neurological disorders.
Dragojlovic N, 2017, Canada	Genetics in Medicine	Cost-effectiveness analysis	Suspected genetic disorders	WES<br>SoC	Hospital	Decision tree	WES: $5,125<br>SoC: $6,138	$18,223	Broad conclusions about the cost-effectiveness of ES should be drawn with caution when relying on studies that use cost or yield assumptions that lie at the extremes of the benchmark ranges.
Radio FC, 2019, Italy	New Genetics and Society	Cost-analysis	Suspected genetic disorders	WES<br>SoC	Hospital	NA	WES: €12,602	NA	We report on the assessment of diagnostic costs referred to a large cohort of patients enrolled in the Bambino Gesù Children's Hospital's "Undiagnosed Patients Program", supporting the cost-effectiveness of

									exome sequencing in a universalistic health care service compared to the traditional multi-step diagnostic workup.
Lavelle TA, 2022, USA	Genetics in Medicine	Cost-effectiveness analysis	Suspected genetic disorders	WGS<br>WES<br>SoC	Health system	Decision tree	WGS: $12,188<br>WES: $10,521<br>SoC: $6,169	$15,048	First-line GS may be the most cost-effective strategy for diagnosing infants with suspected genetic conditions.
Ewans LJ, 2022, Australia	European Journal of Human Genetics	Cost-analysis	Suspected Mendelian disorders	WGS<br>WES	Hospital	NA	WGS: A$267,240<br>WES: A$252,945	A$41,916	Our findings confirm that WGS is the optimal genomic test choice for maximal diagnosis in Mendelian disorders.
Nurchis MC, 2022, Italy	Health Policy	Systematic review and meta-analysis	Suspected genetic disorders	WGS<br>WES<br>SoC	NA	NA	NA	WGS vs WES: I$4073<br>WGS vs SoC: I$6003	WGS could be cost-effective in the diagnostic workup of affected infants and children.

Ontario Health (Quality), 2020, Canada	ONTARIO HEALTH TECHNOLOGY ASSESSMENT SERIES	HTA report	Unexplained Developmental Disabilities Multiple Congenital Anomalies	WGS WES SoC	Health system	Decision tree	WGS: A$7,811 WES: A$6,985 SoC: A$8,783	WES vs SoC: A$11,831 WGS vs WES: dominated	The use of genome-wide sequencing early in the diagnostic pathway (e.g., as a first- or second-tier test) can save on costs and improve diagnostic yields over those of standard testing.
